# Understanding the link between PMN-MDSCs and CXCL8-CXCR1/2 axis in primary myelofibrosis

**DOI:** 10.3389/fcell.2026.1809031

**Published:** 2026-05-15

**Authors:** Rita Campanelli, Stefania Mantovani, Christian Ramirez, Toma Tebaldi, Alessia La Gaipa, Claudia Del Fante, Carlotta Abbà, Adriana Carolei, Paolo Catarsi, Giovanni Barosi, Margherita Massa, Vittorio Rosti

**Affiliations:** 1 Center for the Study of Myelofibrosis, Fondazione IRCCS Policlinico San Matteo, Pavia, Italy; 2 Department of Translational and Clinical Research, Division of Molecular Medicine, Laboratory of Clinical Immunology, Fondazione IRCCS Policlinico San Matteo, Pavia, Italy; 3 Laboratory of RNA and Disease Data Science, Department of Cellular, Computational and Integrative Biology, University of Trento, Povo, Italy; 4 Department of Internal Medicine, Section of Medical Oncology and Hematology, Yale Comprehensive Cancer Center, Yale University School of Medicine, New Haven, CT, United States; 5 Cell Manipulation Laboratory, Immunohaematology and Transfusion Service, Fondazione IRCCS Policlinico San Matteo, Pavia, Italy; 6 General Medicine 2-Center for Systemic Amyloidosis and High-Complexity Diseases, Fondazione IRCCS Policlinico San Matteo, Pavia, Italy

**Keywords:** CXCL12/CXCR4, CXCL8/CXCR1, CXCL8/CXCR2, inflammation, myeloid-derived suppressor cells, primary myelofibrosis

## Abstract

Emergency myelopoiesis in cancer and chronic inflammation leads to the accumulation of myeloid-derived suppressor cells (MDSCs) which localize at tumor sites or areas of chronic inflammation, serving as suppressor cells. Besides reducing the cytotoxic functions of T/NK cells, these cells are major players of the inflammatory process that characterizes the onset and progression of cancer. Inflammation is thought to play a relevant role in the progression of primary myelofibrosis (PMF) and recent studies reported an increased frequency of circulating polymorphonuclear MDSCs (PMN-MDSCs) in these patients, especially in an advanced disease stage. Here, we describe an involvement of the axes CXCL8-CXCR1/2 and, at a lesser extent, CXCL12-CXCR4 in the occurrence of circulating PMN-MDSCs in patients. Our data suggest that, in PMF patients, the persistent inflammatory stimulus promotes the upregulation of CXCR1 expression on PMN-MDSCs (P = 0.024) compared with healthy subjects (HDs). A similar expression of CXCR1 is observed in the circulation of HDs treated with G-CSF (G-HDs) where the egression of MDSCs is related to the chemokine CXCL8. Moreover, the protein levels of CXCR4, an important factor in recruiting MDSCs, are elevated on PMN-MDSCs from PMF (P = 0.02) and G-HDs (P = 0.03) compared to controls, a pattern previously observed in solid cancers. Interestingly, in G-HDs the mobilization of PMN-MDSCs is a consequence of G-CSF stimulation and transient, while in PMF it is possibly mediated by the chronic inflammatory status characterizing the disease. These findings indicate that the overexpression of two distinct membrane receptors plays a prominent role in the biology of MDSCs in PMF.

## Introduction

Primary myelofibrosis (PMF) is a myeloproliferative neoplasm characterized by abnormal proliferation and trafficking of hematopoietic progenitor cells, extramedullary hematopoiesis, variable degrees of bone marrow (BM) fibrosis, splenomegaly and extensive neoangiogenesis in BM and spleen. Inflammation is currently thought to play a relevant role in PMF pathogenesis, as proven by high levels of inflammatory cytokines with prognostic significance, a state of chronic oxidative stress with elevated reactive oxygen species (ROS) together with an abnormal activity of key cells in the immune system (Gangat and Tefferi, 2020; Tefferi, 2023; Koschmieder and Chatain, 2020).

In the context of cancer and chronic inflammation, emergency myelopoiesis is linked to impaired differentiation of myeloid cells. This leads to the accumulation and persistence of immature myeloid-derived suppressor cells (MDSCs), which predominantly localize at tumor sites or areas of chronic inflammation and serve as suppressor cells (Gabrilovich et al., 2012; Meyer et al., 2001; Veglia et al., 2021a). Based on biological function, Gabrilovich and colleagues (Gabrilovich et al., 2012) suggest a two-stage model including: 1) MDSC myelopoiesis in the BM, their mobilization in the blood and secondary lymphatic organs as myeloid cell; 2) their transition and maintenance as MDSC, which takes place at tumor site (Gabrilovich et al., 2012) or sites of chronic inflammation (Meyer et al., 2001). Common features of MDSCs (conventionally divided in polymorphonuclear (PMN)- and monocytic (M)-MDSC subsets) are the strong ability to reduce cytotoxic functions of T/NK cells and a tumor-proangiogenic activity. Moreover, these cells are also considered major players of the inflammatory milieu that characterizes the onset and progression of cancer (Veglia et al., 2021a; Gabrilovich and Nagaraj, 2009; Veglia et al., 2021).

Under physiological conditions, MDSCs and granulocytes are trapped in the BM, until the maturation process is completed, through the membrane expression of CXCR4 and the constitutive production of CXCL12 by osteoblasts and BM stromal cells. In tumor-bearing individuals, there is an increased production of MDSCs and granulocytes together with the impairment of the retention process induced by tumor-derived factors, i.e., granulocyte-colony stimulating factor (G-CSF). G-CSF reduces the production of CXCL12 by BM stromal cells and the expression of CXCR4 on granulocytes, leading to an excessive cell release into the circulation and to their migration towards the tumor site. The same mechanism also drives the egression of MDSCs in BM healthy donors (HDs) after 5-day of G-CSF treatment. Although the precise mechanisms underlying the mobilization of MDSCs are still under investigation, evidence indicates that the recruitment of MDSCs into the tumor microenvironment is due to CXC chemokines and inflammatory cytokines (Veglia et al., 2021a; Groth et al., 2021). Among these, CXCL8–primarily involved in inflammation–and CXCL12–active in both homeostasis and inflammation processes–play central roles (Richardson et al., 1998).

CXCL8 is a key mediator of inflammation and a potent regulator of granulocyte activation and extravasation. Although secreted by different cell types (Ha et al., 2017), CXCL8 is barely detectable in healthy status but could be rapidly induced by various cytokines (IL-1, IL-6, CXCL12, and TNFα) as well as hypoxia, ROS, and other stressors, (Brat et al., 2005; Wald et al., 2013). Upon binding to G protein-coupled receptors (CXCR1 and CXCR2), CXCL8 activates several downstream signaling pathways (Richardson et al., 1998; L'Heureux et al., 1995; Waugh and Wilson, 2008). CXCR1 and CXCR2 are expressed on polymorphonuclear cells, monocytes, endothelia, MDSCs and, although sharing common GPCR signaling pathways and cellular functions, they exert differential functions, i.e., CXCR1 –but not CXCR2– activates phospholipase D and subsequently mediate ROS generation and oxidative burst in neutrophils (Richardson et al., 1998; L'Heureux et al., 1995; Ha et al., 2017).

The overexpression of CXCL8 and/or its receptors has been reported in cancer cells, endothelial cells, infiltrating neutrophils and tumor-associated macrophages. Besides, evidence of overactivation of the CXCL8-CXCR1/2 pathway in cancer have led, in the past two decades, to the development of many therapeutic strategies that are being tested in clinical trials (Xie, 2001; Cheng et al., 2019; Schinke et al., 2015).

Dysregulation of the CXCL12-CXCR4 axis has been implicated in a range of diseases, including autoimmune disorders, immunodeficiencies, developmental abnormalities, and various malignancies; on the other hand, CXCR4-mediated signaling governs critical cellular functions such as proliferation, migration, and stress resistance (Britton et al., 2021). In the last decades, different papers have highlighted the role of CXCR4 in regulating neutrophil retention within the BM and their mobilization during inflammatory responses in murine models. Moreover, CXCR4 plays key roles also in the biology of MDSCs: its expression on MDSCs helps their migration towards tumor cells and tissues and promote their survival and inhibit apoptosis (Qian et al., 2025).

Starting from our previous paper where we demonstrated that in PMF there is a consistent presence of circulating PMN-MDSCs closely linked with disease severity (Campanelli et al., 2024), in this work we have investigated how the CXCL8-CXCR1/2 and CXCL12-CXCR4 signaling pathways influence the mobilization and function of PMN-MDSCs in PMF patients, focusing especially on potential similarities between these patients and G-CSF mobilized healthy individuals.

## Materials and methods

### Patients and healthy subjects

This paper describes a monocentric case-control study; all the subjects were enrolled at Fondazione IRCCS Policlinico San Matteo (Pavia). Demographic, hematologic and molecular characteristics of subjects at the time of blood sampling for MDSC detection are detailed in Supplementary Table S1. Patients with PMF were diagnosed according to the revised 2016 WHO classification (Arber et al., 2016). A total of n = 23 PMF patients, n = 10 healthy subjects (HDs) and n = 12 G-CSF mobilized healthy subjects (G-HDs) were enrolled in this study.

### Flow cytometry analysis and cell sorting

Peripheral blood low-density cells, obtained after gradient centrifugation, were stained with CD15 (ThermoFisher Scientific, Waltham, MA, United States), LOX1 (BioLegend, San Diego, CA, United States), CD11b (Becton Dickinson, Franklin Lakes, NJ, United States) together with CXCR4, CXCR1 and CXCR2 (all BD). Cells were acquired by a flow cytometer (FACSCanto™ II; Becton Dickinson) and analyzed by FACSDiva™ software (Becton Dickinson). PMN-MDSCs were identified as CD11b^+^CD15^+^Lox1^+^ (PMN-MDSCs) and measured as a percentage of the acquired cells; the gating strategy to identify PMN-MDSCs is shown in Supplementary Figure S1. The receptors’ expression was evaluated as percentage of PMN-MDSCs or mean fluorescence intensity (MFI). The specific MFI value was calculated according to appropriate fluorescence minus one (FMO) control and in Supplementary Figure S2 are illustrated representative overlays for fluorescence intensity of CXCR1, CXCR2 and CXCR4 in PMF, G-HDs and HDs. Using the same panel of antibodies, PMN-MDSCs were selected using the BD FACSAria™ Fusion cell sorter and the purity of the cells was tested after the procedure (>95%). We performed the cytofluorimetric analysis in PMF patients (n = 23), HDs (n = 10) and G-HDs (n = 11).

## ELISA

Platelet-poor plasma samples were obtained from PMF patients (n = 23), HDs (n = 7) and G-HDs (n = 6). The levels of CXCL8 and CXCL12 were measured by commercially available ELISA kits (Invitrogen, Carlsbad, CA and R&D Systems Minneapolis, MN, United States, respectively) according to the manufacturer’s instructions.

### RNA sequencing library preparation and sequencing

Total RNA was sorted from purified PMN-MDSCs from PMF patients (n = 8) and G-HDs (n = 7) following standard procedures using RNeasy Plus Mini Kit (Qiagen, Venlo, Netherlands) and RNA quality was assessed using a Fragment Analyzer System (Agilent Technologies, Santa Clara, CA, United States). Libraries were prepared from high-quality RNA using the Illumina® Stranded Total RNA Prep, Ligation with Ribo-Zero Plus kit (Illumina, San Diego, CA, United States), according to the manufacturer’s instructions. Briefly, ribosomal RNA was depleted using DNA probes that bind to rRNA targets, which are subsequently enzymatically digested to produce total RNA depleted of abundant transcripts, followed by RNA fragmentation, first- and second-strand cDNA synthesis, and PCR amplification. Final libraries were quantified using dsDNA Quantification Assay Kit (ThermoFisher) and sized using a Fragment Analyzer System (Agilent Technologies). Sequencing was performed at NGS Core Facility, CIBIO, University of Trento, on NovaSeq 6,000 (Illumina), using a SP flowcell with Paired Ends 100 cycles, adding 1% di PhiX. Sequencing reads were aligned to GRCh38 using STAR and the Gencode 48 gene annotation. Gene expression levels were quantified as fragments per kilobase of transcript per million mapped reads (FPKM).

### Statistical analysis

Statistical analyses were performed using STATISTICA version 8.0 (StatSoft Inc., Tulsa, OK, United States), and in all calculations, a P-value <0.05 was considered statistically significant. Data were tested for normality using the Shapiro–Wilk test. As normality was not met, group comparisons were conducted using the Mann–Whitney U test with Bonferroni correction for multiple comparisons. The Spearman correlation analysis was performed to examine the relationships between the variables under investigation.

## Results

### The CXCL8-CXCR1/2 signaling axis

**FIGURE 1:**
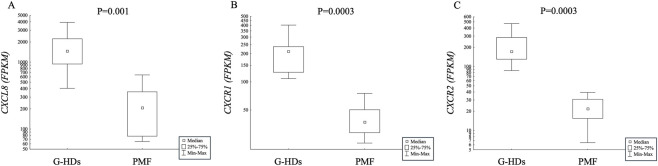
Gene expression of **(A)** CXCLS, **(B)** CXCRI and **(C)** CXCR2 in FACS-selected PMN-MDSCs from the peripheral blood of G-CSF-mobilized healthy subjects (G-HDs; n = 7) and PMF patients (n = 8).

In PMN-MDSCs, the gene expression analysis showed that the *CXCL8* gene was highly expressed (P = 0.001) in G-HDs with respect to PMF patients (Figure 1A). In addition, the gene expression of the two receptors known to bind this chemokine (*CXCR1* and *CXCR2*) was higher and statistically different (P = 0.0003 for both) in PMN-MDSCs isolated from G-HDs than in PMF patients (Figure 1B,C). Analyzing PMF patients according to the mutational status, we found that the expression of all the three above-mentioned genes was higher in G-HDs than in JAK2- or CALR-mutated PMF. Interestingly, *CXCR1* and *CXCR2* genes were higher in G-HDs than in JAK2- (P = 0.048 for both) and CALR-mutated (P = 0.006 for both) patients, while *CXCL8* was significantly different (P = 0.011) with respect to CALR-mutated PMF only (Supplementary Figures S3A–C). While gene expression profiles were comparable between JAK2- and CALR-mutated patients, the possibility remains that a larger cohort could uncover subtle differential expressions. The almost undetectable number of circulating MDSCs made immunoselection not feasible in non-mobilized healthy subjects (HDs) (data not shown).

**FIGURE 2:**
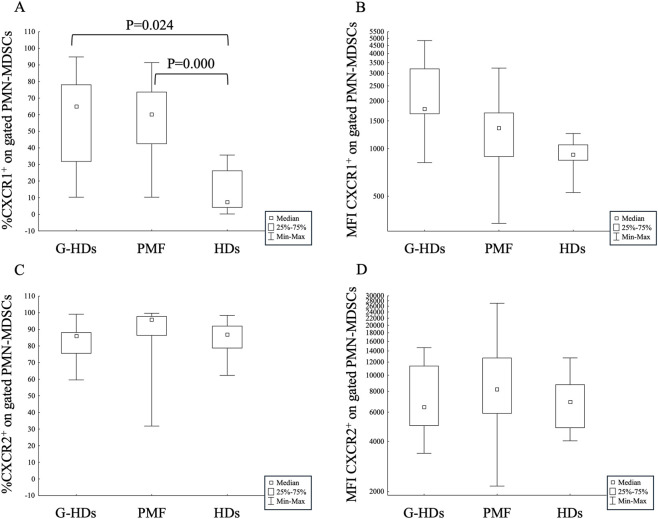
Membrane expression of CXCRI **(A,B)** and CXCR2 **(C,D)** on circulating PMN-MDSCs of G-CSF-mobilized healthy subjects (G-HDs; n = 11), PMF patients (n = 23), and healthy donors (HDs; n = 10). The expression of the receptors was evaluated as percentage **(A,C)** and mean fluorescence intensity (MFI) **(B,D)**.

The receptor analysis showed that CXCR1-expressing PMN-MDSCs were significantly higher both in PMF (P = 0.000) and G-HDs (P = 0.024) with respect to healthy subjects (HDs) but comparable between PMF and G-HDs (Figure 2A). Despite being elevated, CXCR2-expressing PMN-MDSCs were not different in PMF, G-HDs and HDs (Figure 2C). The mean fluorescence intensity (MFI) of CXCR1 on the membrane of PMN-MDSCs, although higher in G-HDs with respect to PMF and HDs, was not statistically different (Figure 2B), while the MFI of CXCR2 had comparable levels in the three groups analyzed (Figure 2D). The receptors’ analysis in PMF patients according to the mutational status only highlighted an increased percentage of CXCR1-expressing PMN-MDSCs in G-HDs (P = 0.032), JAK2- (P = 0.0015) and CALR-mutated patients (P = 0.0012) with respect to HDs (Supplementary Figure S4A). No statistical differences were observed in the other parameters studied (Supplementary Figures S4B–D).

We found no statical difference between patients on-therapy and off-therapy for all the parameters related to the axis CXCL8-CXCR1/2 and CXCL12-CXCR4 (data not shown).

The plasmatic levels of CXCL8 were not statistically different among G-HDs (n = 6; median 1.1 pg/mL, range 0.4–1.9), PMF (n = 23; median 2.2 pg/mL, range 0.4–8.3) and HDs (n = 7; median 1.7 pg/mL, range 1.1–2.5); in addition, we found no correlations between CXCL8 plasmatic levels and the expression of membrane receptors in all the groups (data not shown).

### The CXCL12-CXCR4 signaling axis

In immune-selected PMN-MDSCs, the expression of the *CXCL12* gene was undetectable both in G-HDs and PMF suggesting that the chemokine is mainly produced by other cell types.

**FIGURE 3:**
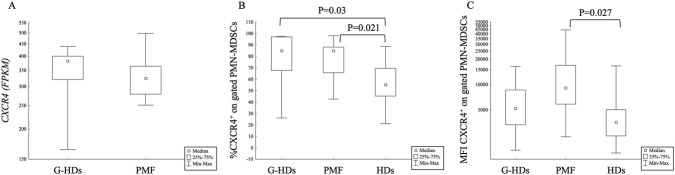
**(A)** Gene expression of CXCR4 in FACS-selected PMN-MDSCs obtained from G-CSF-mobilized healthy subjects (G-HDs; n = 7) and PMF patients (n = 8). **(B,C)** Membrane expression of CXCR4 on PMN-MDSCs of G-CSF-mobilized healthy subjects (G-HDs; n = 11), PMF patients (n = 23), and healthy donors (HDs; n = 10). The expression of the receptor was evaluated as percentage **(B)** and mean fluorescence intensity (MFI) **(C)**.

The expression of the *CXCR4* gene was detectable, although not statistically different, in PMN-MDSCs obtained from G-HDs and PMF (Figure 3A) and, considering the mutational status of patients, we also observed a similar expression of *CXCR4* in JAK2-, CALR-mutated PMF and G-HDs (Supplementary Figure S5A).

We then analyzed the membrane expression of the receptor, and we found that CXCR4^+^ PMN-MDSCs were significantly higher in G-HDs (P = 0.030) and PMF (P = 0.021) than in HDs (Figure 3B) while the MFI was only higher in PMF (P = 0.027) than in HDs (Figure 3C). Similarly, the membrane analysis of CXCR4 (percentage of positive cells and MFI) in JAK2- or CALR-mutated PMF patients highlighted that there was an increase of CXCR4-expressing PMN-MDSCs in G-HDs (P = 0.04), and CALR-mutated PMF (P = 0.012) compared to HDs (Supplementary Figure S5B). As shown in Supplementary Figure S5C, the membrane expression of CXCR4 was more difficult to interpret because this receptor exhibited a wide range of fluorescence intensities in all the subjects evaluated. The expression of CXCR4 in CALR-mutated patients was higher (P = 0.04) than in HDs; however, a larger sample size would be required to clarify this finding.

The evaluation of CXCL12 revealed an augmented plasmatic concentration of the chemokine in PMF patients (n = 22; median 2,561 pg/mL, range 1,576–4,261) with respect to G-HDs (n = 7; median 1,422 pg/mL; range 1,195–2,234) and HDs (n = 7; median 1776 pg/mL; range 1,273–2,732) with p-values 0.0003 and 0.004, respectively. No correlations were found between plasmatic levels of the chemokine and CXCR4 (data not shown).

## Discussion

In this study, we showed that in PMF patients, the percentage of circulating MDSCs is related with the expression of CXCR1, one of the receptors of the chemokine CXCL8.

Previously, we have documented that the polymorphonuclear subset of MDSCs (PMN-MDSCs) was significantly increased and consistent in PMF patients, strongly associated with genotype, clinical and biological parameters of the disease as well as positively correlated with the disease progression (Campanelli et al., 2024). Moreover, it was shown that the plasmatic concentration of CXCL8 is increased in PMF, independently from the mutational status, and that higher levels of CXCL8 in myeloproliferative neoplasms (MPNs) are linked to poorer outcomes and decreased overall survival (Tefferi et al., 2011; Øbro et al., 2020). Despite these observations, the precise functions of CXCL8 and its related receptors in myelofibrosis remain unclear.

Our findings show that CXCR1 had a higher gene expression in G-HDs than in PMF but a comparable protein amount on the cell membrane of PMN-MDSCs isolated from the two groups of subjects. Interestingly, there was a significant difference between G-HDs, PMF and HDs suggesting a shared mechanism leading to the mobilization process of PMN-MDSCs from the BM. A possible explanation for the “discrepancy” between CXCR1 gene and protein expression in G-HDs and PMF is that G-CSF stimulation may induce a rapid transcriptional upregulation that is not fully translated into the corresponding membrane protein. However, for a potential interpretation of this discrepancy two important points should be taken into account. First, we could not analyze the CXCR1/2 RNA levels of non-mobilized HDs, preventing us to rule out the possibility that - although lower than in G-HDs - mRNA levels in PMF patients were higher than non-mobilized healthy subjects, therefore resulting in increased levels of the membrane receptor, at least for the CXCR1. The impossibility of analyzing CXCR1/2 gene expression in non-mobilized HDs represent a major limitation that, however, can not be overcome, due to the very low frequency of MDSCs in these subjects. Second, MDSCs of G-CSF mobilized subjects’ egress from a healthy bone marrow under the effect of a pharmacologic stimulus, persist in the blood as long as the stimulus occurs, quickly disappearing when it ceases. On the contrary, circulating MDSCs in PMF patients egress from a pathologic bone marrow characterized by an inflammatory microenvironment that can affect the normal physiology of MDSCs that, in fact, persist for years in the circulation. Being the result of a pathologic status, these MDSCs could be characterized by the dysregulation of one or more physiologic processes mediated by epigenetic mechanisms, including altered post translational regulations, such as mRNA increased half-life, and/or internalization rates of CXCR on the cell surface.

On the contrary, *CXCR2* was higher in G-HDs than in PMF as gene expression, but the membrane protein was comparable among G-HDs, PMF and HDs. This result was unexpected given the established role of this receptor in the mobilization process of myeloid cells. It is likely that assessment of *CXCR1* and *CXCR2* gene expression in non-mobilized HDs could have helped in clarifying this finding however, the scarce number of circulating PMN-MDSCs in HDs prevented us from isolating this population and performing gene expression evaluation.

Besides its role in the regulation of MDSC mobilization, the CXCL8-CXCR1 axis could also be involved in the onset/progression of the fibrotic process that is a feature of the bone marrow of PMF patients, and a determinant of hematopoietic failure observed in the progression of the disease. Although a definitive mechanism for the onset of bone marrow fibrosis in PMF is not completely known up to now, it cannot be excluded that a reactive fibrosis to the inflamed microenvironment could be, in part, one of the components of a multi factorial pathogenetic process that end-up in the generation of bone marrow fibrosis. This could be determined directly by CXCL8 or other(s) cytokines eventually produced by MDSCs or in an indirect way by other determinants of the inflammatory milieu that characterizes the disease and that is fueled by MDSCs themselves (Chatain et al., 2020).

In summary, the overexpression of CXCR1 and its relationship with the number of circulating PMN-MDSCs in PMF indicates an involvement of the CXCL8-CXCR1/2 axis in the mobilization process that leads to the release of these cells from the BM and/or their endurance in the circulation of patients. This mechanism mirrors what happens in healthy donors treated with G-CSF where, besides inducing stem cell mobilization, G-CSF also promotes the egression of MDSCs, and recent studies have suggested that the amount of MDSCs in the graft has an important role in the post-transplant outcome. Moreover, G-CSF treatment upregulates CXCR1 on the membrane of PMN-MDSCs (*personal observations*). It should be noted that, in G-CSF-stimulated healthy donors, this effect is likely transient as MDSCs are rarely detectable in the peripheral blood of non-mobilized healthy individuals and post-transplant analyzes in multiple myeloma have shown that MDSCs become undetectable 12 months after transplantation (Tyrinova et al., 2024). In contrast, PMF is a long-term chronic condition in which PMN-MDSCs persist in the circulation and their number increases with the disease progression. Two non-exclusive mechanisms can explain this phenomenon: 1) an epigenetic mechanism that modulate the *CXCR1* gene expression, ensuring an elevated protein expression; 2) a consistent factor, such as the inflammatory environment that characterizes PMF patients, that promotes CXCR1 upregulation, modulating the mobilization and/or peripheral endurance of patients.

Several studies have identified CXCR4 as an important mediator of MDSC recruitment, regulated by an autocrine production of cytokines (Obermajer et al., 2011; Liu et al., 2012), while its ligand CXCL12 mediates the reduction of MDSC apoptosis (Jiang et al., 2019). Interestingly, the increased expression of CXCR4 on PMN-MDSCs in PMF and G-HDs compared with CTRLs is similar to what was observed in MDSCs circulating in patients with solid tumors, where CXCR4 is frequently found to be increased (Groth et al., 2021). Surprisingly, our result contrasts with what has been observed in circulating CD34^+^ stem cells, in which CXCR4 membrane expression is reduced (Rosti et al., 2007) and the CXCR4 promoter hypermethylated (Bogani et al., 2008). Although a definitive explanation of this discrepancy is not available, we hypothesize that it may reflect the distinct maturation stages of MDSCs compared with hematopoietic stem cells. Furthermore, the role of CXCR4 in MDSC biology remains unclear, due to the dynamic surface expression and functional instability of the receptor, characteristics that can influence the protein expression even when the gene expression remains stable. This consideration is particularly relevant in chronic diseases lasting decades, such as PMF, where MDSCs can continuously exit from the BM and/or remain in the circulation due to dysregulated apoptotic processes. Additional studies are required to identify the molecular features associated with apoptosis or cell cycle stages in PMN-MDSCs derived from patients with PMF, especially those involving CXCR4.

Taken together, our results shed new knowledge on the involvement of an aberrant CXCR1 expression in PMN-MDSC mobilization in PMF patients. Further studies on a larger number of patients should reinforce the link between CXCR1 dysregulation and MDSC mobilization, potentially establishing CXCR1 as a therapeutic target in PMF patients (as already demonstrated in ongoing clinical trials applied to hematologic and solid tumors) aiming to reduce the presence of circulating PMN-MDSCs that contribute to the disease worsening. This could be achieved both favoring their differentiation into mature PMN cells and/or lowering their survival rate and/or inhibiting their ability to sustain the inflammatory process.

## Data Availability

The raw data supporting the conclusions of this article will be made available by the authors, without undue reservation.

## References

[B1] Arber D. A. Orazi A. Hasserjian R. Thiele J. Borowitz M. J. Le Beau M. M. (2016). The 2016 revision to the world health Organization classification of myeloid neoplasms and acute leukemia. Blood 127 (20), 2391–2405. 10.1182/blood-2016-03-643544 27069254

[B2] Bogani C. Ponziani V. Guglielmelli P. Desterke C. Rosti V. Bosi A. (2008). Hypermethylation of CXCR4 promoter in CD34+ cells from patients with primary myelofibrosis. Stem Cells 26 (8), 1920–1930. 10.1634/stemcells.2008-0377 18511598

[B3] Brat D. J. Bellail A. C. Van Meir E. G. (2005). The role of interleukin-8 and its receptors in gliomagenesis and tumoral angiogenesis. Neuro Oncol. 7 (2), 122–133. 10.1215/S1152851704001061 15831231 PMC1871893

[B4] Britton C. Poznansky M. C. Reeves P. (2021). Polyfunctionality of the CXCR4/CXCL12 axis in health and disease: implications for therapeutic interventions in cancer and immune‐mediated diseases. FASEB J. 35 (4), e21260. 10.1096/fj.202001273r 33715207

[B5] Campanelli R. Carolei A. Catarsi P. Abbà C. Boveri E. Paulli M. (2024). Circulating polymorphonuclear myeloid-derived suppressor cells (PMN-MDSCs) have a biological role in patients with primary myelofibrosis. Cancers 16 (14), 2556. 10.3390/cancers16142556 39061196 PMC11275082

[B6] Chatain N. Koschmieder S. Jost E. (2020). Role of inflammatory factors during disease pathogenesis and stem cell transplantation in myeloproliferative neoplasms. Cancers 12 (8), 2250. 10.3390/cancers12082250 32806517 PMC7463735

[B7] Cheng Y. Ma X. Wei Y. Wei X. (2019). Potential roles and targeted therapy of the CXCLs/CXCR2 axis in cancer and inflammatory diseases. Biochim. Biophys. Acta Rev. Cancer 1871 (2), 289–312. 10.1016/j.bbcan.2019.01.005 30703432

[B8] Gabrilovich D. I. Nagaraj S. (2009). Myeloid-derived-suppressor cells as regulators of the immune system. Nat. Rev. Immunol. 9 (3), 162–174. 10.1038/nri2506 19197294 PMC2828349

[B9] Gabrilovich D. I. Ostrand-Rosenberg S. Bronte V. (2012). Coordinated regulation of myeloid cells by tumours. Nat. Rev. Immunol. 12 (4), 253–268. 10.1038/nri3175 22437938 PMC3587148

[B10] Gangat N. Tefferi A. (2020). Myelofibrosis biology and contemporary management. Br. J. Haematol. 191 (2), 152–170. 10.1111/bjh.16576 32196650

[B11] Groth C. Weber R. Lasser S. Özbay F. G. Kurzay A. Petrova V. (2021). Tumor promoting capacity of polymorphonuclear myeloid‐derived suppressor cells and their neutralization. Intl J. Cancer 149 (9), 1628–1638. 10.1002/ijc.33731 34224592

[B12] Ha H. Debnath B. Neamati N. (2017). Role of the CXCL8-CXCR1/2 axis in cancer and inflammatory diseases. Theranostics 7 (6), 1543–1588. 10.7150/thno.15625 28529637 PMC5436513

[B13] Jiang K. Li J. Zhang J. Wang L. Zhang Q. Ge J. (2019). SDF-1/CXCR4 axis facilitates myeloid-derived suppressor cells accumulation in osteosarcoma microenvironment and blunts the response to anti-PD-1 therapy. Int. Immunopharmacol. 75, 105818. 10.1016/j.intimp.2019.105818 31437795

[B14] Koschmieder S. Chatain N. (2020). Role of inflammation in the biology of myeloproliferative neoplasms. Blood Rev. 42, 100711. 10.1016/j.blre.2020.100711 32505517

[B15] L'Heureux G. P. Bourgoin S. Jean N. McColl S. R. Naccache P. H. (1995). Diverging signal transduction pathways activated by Interleukin-8 and related chemokines in human neutrophils: interleukin-8, but not NAP-2 or GROα, stimulates phospholipase D activity. Blood 85 (2), 522–531. 10.1182/blood.V85.2.522.522 7812007

[B16] Liu Y. Lai L. Chen Q. Song Y. Xu S. Ma F. (2012). MicroRNA-494 is required for the accumulation and functions of tumor-expanded myeloid-Derived suppressor cells *via* targeting of PTEN. J. Immunol. 188 (11), 5500–5510. 10.4049/jimmunol.1103505 22544933

[B17] Meyer M. Hensbergen P. J. Raaij-Helmer E. M. H. V. D. Brandacher G. Margreiter R. Heufler C. (2001). Cross reactivity of three T cell attracting murine chemokines stimulating the CXC chemokine receptor CXCR3 and their induction in cultured cells and during allograft rejection. Eur. J. Immunol. 31 (8), 2521–2527. 10.1002/1521-4141(200108)31:8<2521::aid-immu2521>3.0.co;2-q 11500837

[B18] Obermajer N. Muthuswamy R. Odunsi K. Edwards R. P. Kalinski P. (2011). PGE2-Induced CXCL12 production and CXCR4 expression controls the accumulation of human MDSCs in ovarian cancer environment. Cancer Res. 71 (24), 7463–7470. 10.1158/0008-5472.CAN-11-2449 22025564 PMC4993027

[B19] Øbro N. F. Grinfeld J. Belmonte M. Irvine M. Shepherd M. S. Rao T. N. (2020). Longitudinal cytokine profiling identifies GRO-α and EGF as potential biomarkers of disease progression in essential thrombocythemia. Hemasphere 4 (3), e371. 10.1097/HS9.0000000000000371 32647796 PMC7306314

[B20] Qian J. Ma C. Waterbury Q. T. Zhi X. Moon C. S. Tu R. (2025). A CXCR4 partial agonist improves immunotherapy by targeting polymorphonuclear myeloid-derived suppressor cells and cancer-driven granulopoiesis. Cancer Cell 43 (8), 1512. 10.1016/j.ccell.2025.06.006 40578360 PMC12233206

[B21] Richardson R. M. Ali H. Pridgen B. C. Haribabu B. Snyderman R. (1998). Multiple Signaling Pathways of Human Interleukin-8 receptor A. Independent regulation of phosphorylation. J. Biol. Chem. 273 (17), 10690–10695. 10.1074/jbc.273.17.10690 9553132

[B22] Rosti V. Massa M. Vannucchi A. M. Bergamaschi G. Campanelli R. Pecci A. (2007). The expression of CXCR4 is down-regulated on the CD34+ cells of patients with myelofibrosis with myeloid metaplasia. Blood Cells Mol. Dis. 38 (3), 280–286. 10.1016/j.bcmd.2007.01.003 17350297

[B23] Schinke C. Giricz O. Li W. Shastri A. Gordon S. Barreyro L. (2015). IL8-CXCR2 pathway inhibition as a therapeutic strategy against MDS and AML stem cells. Blood 125 (20), 3144–3152. 10.1182/blood-2015-01-621631 25810490 PMC4432009

[B24] Tefferi A. (2023). Primary myelofibrosis: 2023 update on diagnosis, risk-stratification, and management. Am. J. Hematol. 98 (5), 801–821. 10.1002/ajh.26857 36680511

[B25] Tefferi A. Vaidya R. Caramazza D. Finke C. Lasho T. Pardanani A. (2011). Circulating interleukin (IL)-8, IL-2R, IL-12, and IL-15 levels are independently prognostic in primary myelofibrosis: a comprehensive cytokine profiling study. J. Clin. Oncol. 29 (10), 1356–1363. 10.1200/JCO.2010.32.9490 21300928

[B26] Tyrinova T. Batorov E. Aristova T. Ushakava G. Sizikova S. Denisova V. (2024). Decreased circulating myeloid-derived suppressor cell count at the engraftment is one of the risk factors for multiple myeloma relapse after autologous hematopoietic stem cell transplantation. Heliyon 10 (5), e26362. 10.1016/j.heliyon.2024.e26362 38434301 PMC10907647

[B27] Veglia F. Hashimoto A. Dweep H. Sanseviero E. De Leo A. Tcyganov E. (2021a). Analysis of classical neutrophils and polymorphonuclear myeloid-derived suppressor cells in cancer patients and tumor-bearing mice. J. Exp. Med. 218 (4), e20201803. 10.1084/jem.20201803 33566112 PMC7879582

[B28] Veglia F. Sanseviero E. Gabrilovich D. I. (2021b). Myeloid-derived suppressor cells in the era of increasing myeloid cell diversity. Nat. Rev. Immunol. 21 (8), 485–498. 10.1038/s41577-020-00490-y 33526920 PMC7849958

[B29] Wald O. Shapira O. M. Izhar U. (2013). CXCR4/CXCL12 axis in non small cell lung Cancer (NSCLC) pathologic roles and therapeutic potential. Theranostics 3 (1), 26–33. 10.7150/thno.4922 23382783 PMC3563078

[B30] Waugh D. J. J. Wilson C. (2008). The interleukin-8 pathway in cancer. Clin. Cancer Res. 14 (21), 6735–6741. 10.1158/1078-0432.CCR-07-4843 18980965

[B31] Xie K. (2001). Interleukin-8 and human cancer biology. Cytokine Growth Factor Rev. 12 (4), 375–391. 10.1016/S1359-6101(01)00016-8 11544106

