# Supplementary material for: Understanding the link between PMN-MDSCs and CXCL8-CXCR1/2 axis in primary myelofibrosis
Source: Front Cell Dev Biol. 2026 May 15;14:1809031. doi: 10.3389/fcell.2026.1809031 (PMC13219034; doi:10.3389/fcell.2026.1809031)

FIGURE S5. **(A)** Gene expression of *CXCR4* in FACS-selected PMN-MDSCs obtained from G-CSF-mobilized healthy subjects (G-HDs; n= 7) and PMF patients JAK2-mutated (n= 3) or CALR-mutated (n= 5). **(B-C)** Membrane expression of CXCR4 on PMN-MDSCs of G-CSF-mobilized healthy subjects (G-HDs; n= 11), PMF patients JAK2-mutated (n= 16) or CALR-mutated (n= 7) and healthy donors (HDs; n= 10). The expression of the receptor was evaluated as percentage **(B)** and mean fluorescence intensity (MFI) **(C)**.

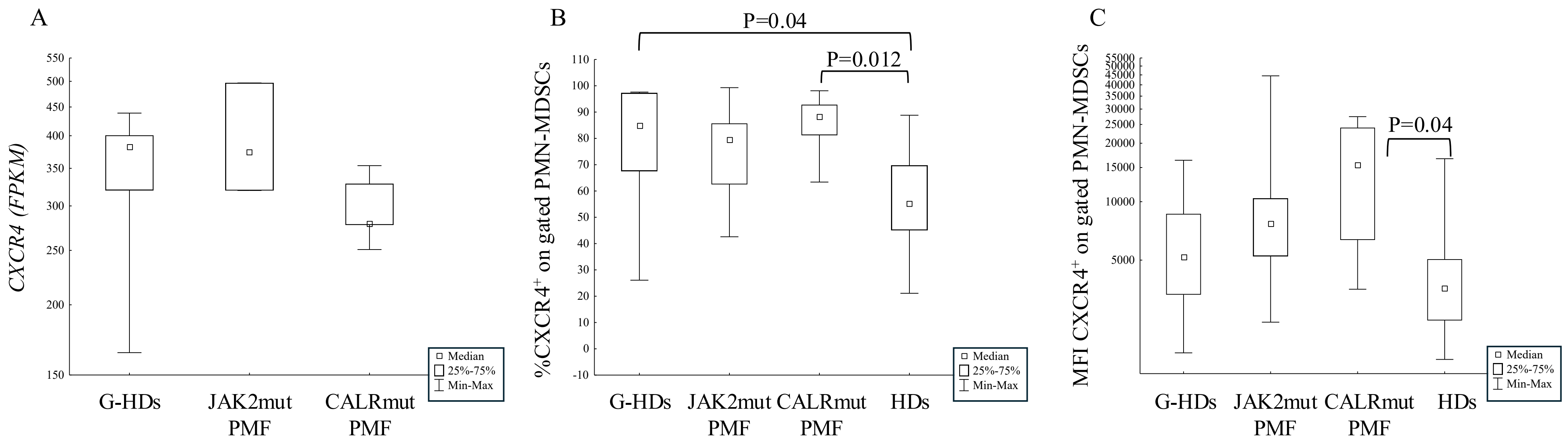

Supplement: Supplementary file 1 [file Image5.pdf]
