# Supplementary material for: Understanding the link between PMN-MDSCs and CXCL8-CXCR1/2 axis in primary myelofibrosis
Source: Front Cell Dev Biol. 2026 May 15;14:1809031. doi: 10.3389/fcell.2026.1809031 (PMC13219034; doi:10.3389/fcell.2026.1809031)
Supplement: Supplementary file 2 [file Table1.pdf]

TABLE S1. The upper part of the table illustrates demographic and therapy-related data of patients with primary myelofibrosis (PMF), healthy subjects (HDs) and G-CSF-mobilized healthy subjects (G-HDs) at the time of blood sampling for myeloid-derived suppressor cell (MDSC) detection; the lower section of the table illustrates the clinical–hematological characteristics of PMF patients.

	**Primary Myelofibrosis (PMF)**	**Healthy Subjects (HDs)**	**G-CSF-mobilized HDs (G-HDs)**
Number of subjects	23	10	12
Age (years), median (range)	57 (41-75)	48 (29-64)	33 (21-54)
Out of therapy, number (percent)	9 (39%)	10 (100%)	12 (100%)
In therapy, number			
-hydroxycarbamide	11 (48%)		
-JAK-inhibitors	3 (13%)		
**<u>PMF clinical–hematological characteristics</u>**			
Hemoglobin (g/L), median (range)	128 (86-157)		
White-blood cell count ($\times 10^9$/L), median (range)	6.9 (3.6-11.5)		
Platelet count ($\times 10^9$/L), median (range)	498 (193-977)		
LDH (mU/mL), median (range)	311 (174-770)		
Blasts, number of PMF (percent)			
- 0	23 (100%)		
- 1	-		
- ≥2	-		
$CD34^+$ absolute number/μL, median (range)	6.8 (0.59-78.1)		
DIPPS, number of PMF (percent)			
- low risk	15 (65.2)		
- intermediate -1	7 (30.4)		
- intermediate -2	1 (4.4)		
