# Supplementary material for: Understanding the link between PMN-MDSCs and CXCL8-CXCR1/2 axis in primary myelofibrosis
Source: Front Cell Dev Biol. 2026 May 15;14:1809031. doi: 10.3389/fcell.2026.1809031 (PMC13219034; doi:10.3389/fcell.2026.1809031)

FIGURE S4. Membrane expression of CXCR1 **(A, B)** and CXCR2 **(C, D)** on PMN-MDSCs of G-CSF-mobilized healthy subjects (G-HDs; n= 11), PMF patients JAK2-mutated (n= 16) or CALR-mutated (n= 7), and healthy donors (HDs; n= 10). The expression of the receptors was evaluated as percentage **(A, C)** and mean fluorescence intensity (MFI) **(B, D)**.

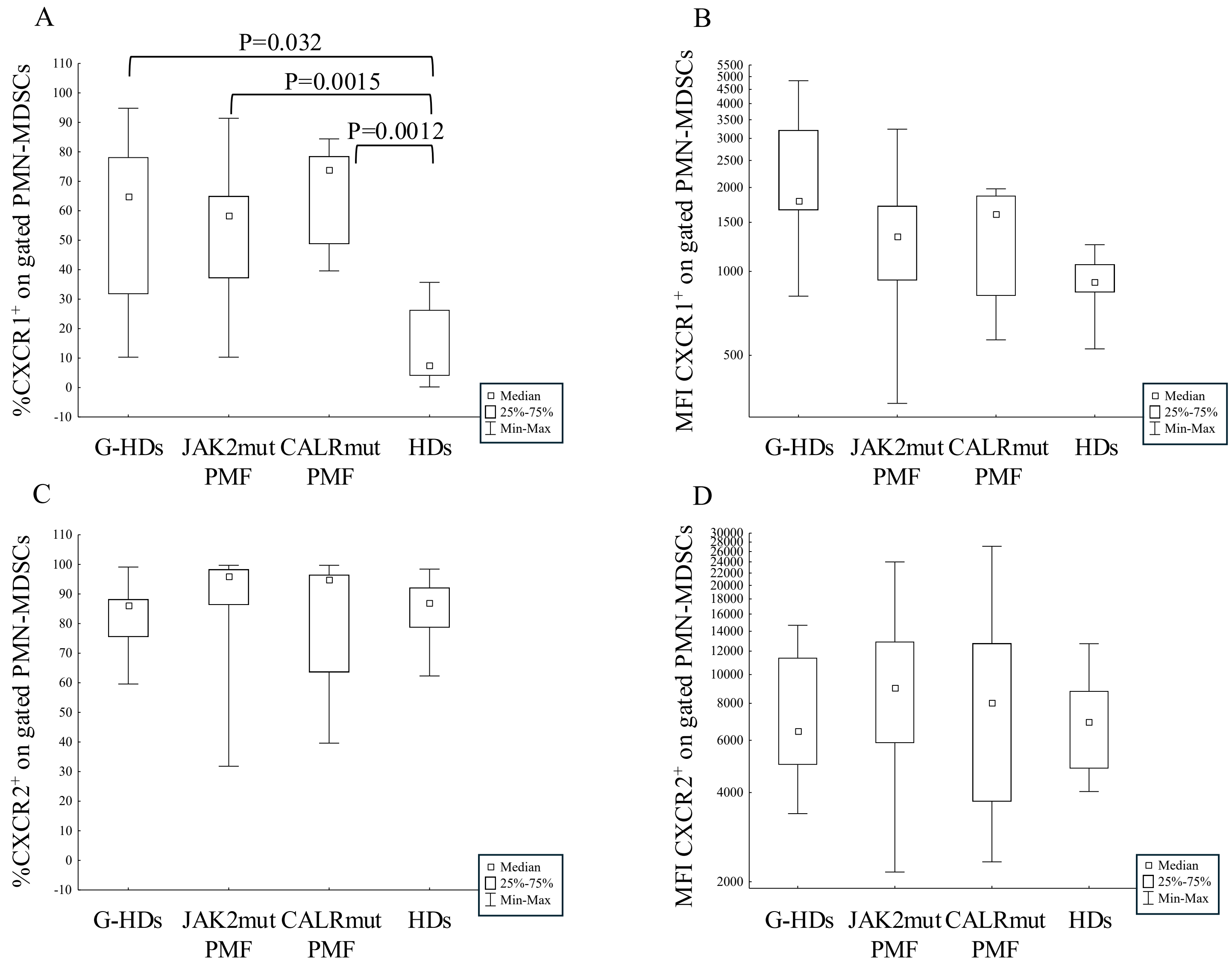

Supplement: Supplementary file 3 [file Image4.pdf]
