# Supplementary material for: Understanding the link between PMN-MDSCs and CXCL8-CXCR1/2 axis in primary myelofibrosis
Source: Front Cell Dev Biol. 2026 May 15;14:1809031. doi: 10.3389/fcell.2026.1809031 (PMC13219034; doi:10.3389/fcell.2026.1809031)

FIGURE S2. Overlays showing the fluorescence intensity of CXCR1, CXCR2, CXCR4 in one representative sample for each group of subjects evaluated in the paper: primary myelofibrosis (PMF), G-CSF mobilized healthy subjects (G-HDs) and healthy subjects (HDs).

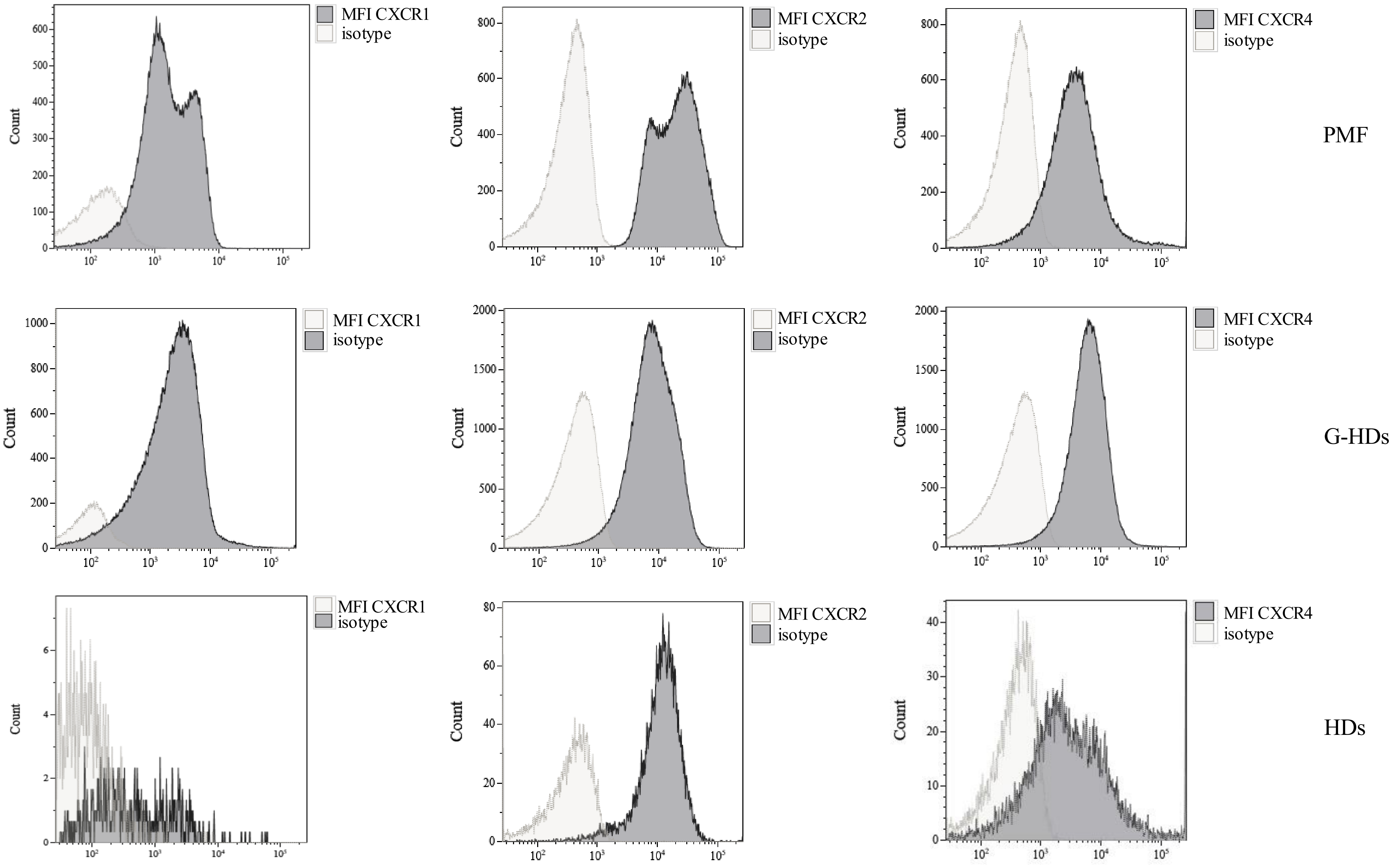

Supplement: Supplementary file 4 [file Image2.pdf]
