# Supplementary material for: Understanding the link between PMN-MDSCs and CXCL8-CXCR1/2 axis in primary myelofibrosis
Source: Front Cell Dev Biol. 2026 May 15;14:1809031. doi: 10.3389/fcell.2026.1809031 (PMC13219034; doi:10.3389/fcell.2026.1809031)

FIGURE S3. Gene expression of **(A)** *CXCL8*, **(B)** *CXCR1* and **(C)** *CXCR2* in FACS-selected PMN-MDSCs from the peripheral blood of G-CSF-mobilized healthy subjects (G-HDs; n= 7) and PMF patients JAK2-mutated (n= 3) or CALR-mutated (n= 5).

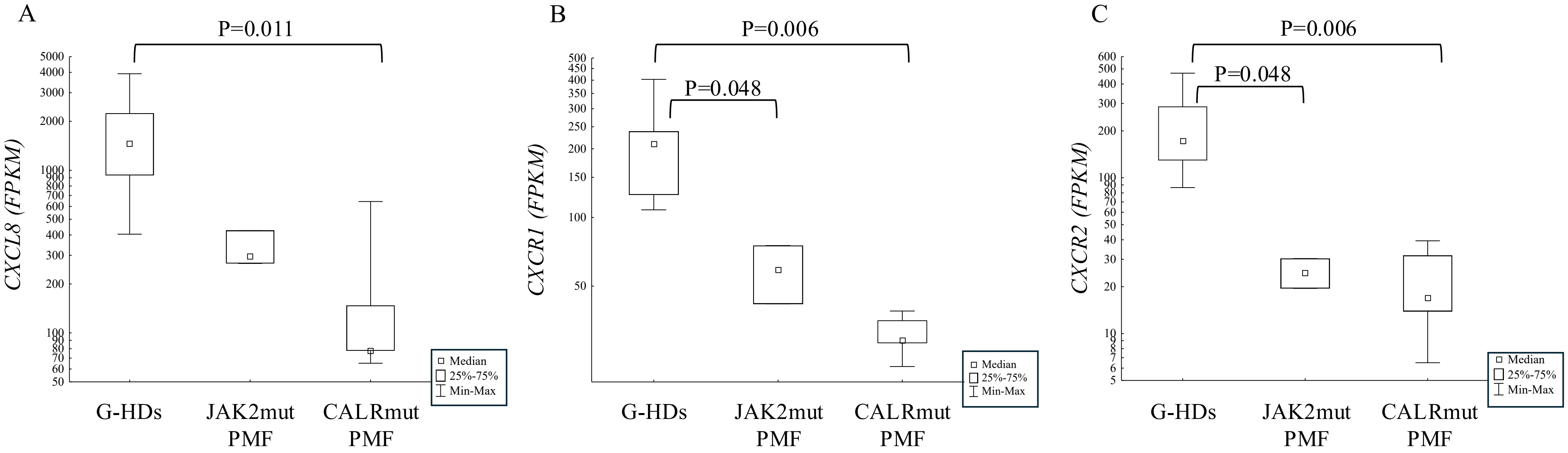

Supplement: Supplementary file 5 [file Image3.pdf]
