# Supplementary material for: Understanding the link between PMN-MDSCs and CXCL8-CXCR1/2 axis in primary myelofibrosis
Source: Front Cell Dev Biol. 2026 May 15;14:1809031. doi: 10.3389/fcell.2026.1809031 (PMC13219034; doi:10.3389/fcell.2026.1809031)

FIGURE S1. Gating strategy to identify PMN-MDSCs (CD11b^{+}CD15^{+}LOX1^{+}) in peripheral blood of patients with primary myelofibrosis (PMF), G-CSF mobilized healthy subjects (G-HDs) and healthy subjects (HDs) by flow cytometry.

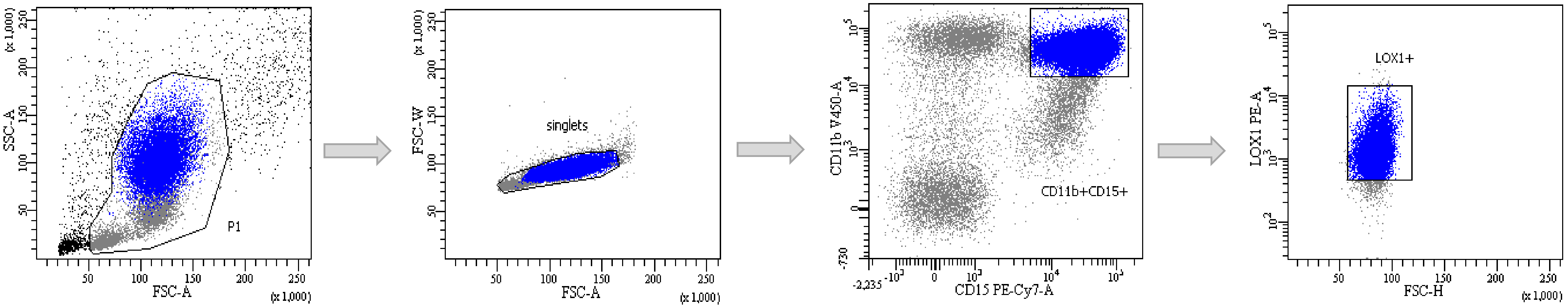

Supplement: Supplementary file 6 [file Image1.pdf]
